# Clonal hematopoiesis associates with prevalent and incident cardiometabolic disease in a cardiac catheterization cohort

**DOI:** 10.1371/journal.pone.0339491

**Published:** 2026-02-10

**Authors:** Jessica A. Regan, Lydia Coulter Kwee, Navid A. Nafissi, Alexander G. Bick, William E. Kraus, Pradeep Natarajan, Siddhartha Jaiswal, Svati H. Shah

**Affiliations:** 1 Division of Cardiology, Department of Medicine, Duke University, Durham, North Carolina, United States of America; 2 Duke Molecular Physiology Institute, Durham, North Carolina, United States of America; 3 Division of Genetic Medicine, Department of Medicine, Vanderbilt University School of Medicine, Nashville, Tennessee, United States of America; 4 Center for Genomic Medicine and Cardiovascular Research Center, Massachusetts General Hospital, Boston, Massachusetts, United States of America; 5 Program in Medical & Population Genetics, Broad Institute of Harvard & MIT, Cambridge, Massachusetts, United States of America; 6 Department of Medicine, Harvard Medical School, Boston, Massachusetts, United States of America; 7 Department of Pathology, Stanford University School of Medicine, Stanford, California, United States of America; Longgang Otorhinolaryngology Hospital & Shenzhen Key Laboratory of Otorhinolaryngology, Shenzhen Institute of Otorhinolaryngology, CHINA

## Abstract

**Background:**

Clonal hematopoiesis of indeterminate potential (CHIP) is the age-related presence of expanded somatic clones secondary to leukemogenic driver mutations and is associated with cardiovascular (CV) disease and mortality. We sought to evaluate relationships between CHIP with cardiometabolic diseases and incident outcomes in high-risk individuals.

**Methods:**

CHIP genotyping was performed in 8469 individuals referred for cardiac catheterization at Duke University (CATHGEN study) to identify variants present at a variant allele fraction (VAF) ≥2%. Associations were tested among any CHIP variant, large CHIP clones (VAF > 10%) and individual CHIP genes with prevalent cardiometabolic traits. Cox proportional hazard models tested CHIP associations with time-to-overall mortality and Fine-Gray analyses tested CHIP associations with incident cardiovascular outcomes.

**Results:**

We identified 463 CHIP variants in 427 individuals (5.0%) of which 268 (3.2%) harbored large CHIP clones. CHIP and large CHIP were associated with lower odds of obesity (OR 0.79 [95% CI 0.65–0.98], p = 0.03; OR 0.76 [95% CI 0.57–0.99], p = 0.04, respectively). CHIP was associated with prevalent heart failure (HF, OR 1.25 [95% CI 1.01–1.55], p = 0.04; especially for non-*DNMT3A* CHIP (OR 1.38 [95% CI 1.04–1.82], p = 0.02). CHIP was also associated with incident events: Non-*DNMT3A* CHIP was associated with increased risk of time-to-HF hospitalization (HR 1.29 [95% CI 1.02–1.63], p = 0.03).

**Conclusions:**

In high-risk individuals referred for cardiac catheterization, large CHIP and non-*DNTM3A* CHIP were associated with obesity, prevalent HF, incident CV events. These findings strengthen the importance of CHIP as a biomarker for CV disease and highlight the contributing risk of large CHIP clones and non-*DNMT3A* CHIP variants.

## Introduction

Genetic epidemiology studies have identified hundreds of variants associated with cardiovascular disease (CVD); however, these variants typically have small effect sizes and incompletely predict CVD risk [1]. While work has focused on germline mutations, which occur with formation of the embryo and are static over the lifetime, somatic mutations have increasingly been shown to contribute to CVD. Inherent in cancers, somatic mutations have been identified in diverse non-tumor tissues including in the atria of patients with atrial fibrillation (AF) [2].

Clonal hematopoiesis (CH) is the age-related expansion of somatic clones in hematopoietic stem cells (HSCs). CH of indeterminate potential (CHIP) can be detected in peripheral blood DNA and is defined as having a clone size (variant allele fraction [VAF]) ≥2% in genes associated with the development of myeloid malignancies [3–5]. The most frequently mutated CHIP genes are *DNMT3A*, *TET2* and *ASXL1*, where mutant cells have a competitive proliferative advantage over native HSCs. Germline contributors to acquisition of CHIP have been identified [6–8], and smoking has been associated with increased odds of *ASXL1*-mutated CHIP [6].

The presence of CHIP is associated with increased risk of overall mortality, which appears to be driven by CVD events more so than by malignancy-related mortality. CHIP is associated with increased risk of incident coronary artery disease (CAD) and accelerated atherosclerosis, with heightened levels of inflammation in both clinical and pre-clinical models [9,10]. Large CHIP clones (VAF > 10%) in *DNMT3A* and *TET2* contribute greater risk for incident CVD [11]. CH is also associated with incident heart failure (HF) [8,12,13] including incident heart failure with preserved ejection fraction (HFpEF) [14,15], as well as AF [8,16–18]. Variants in particular CHIP genes, have been identified as drivers of phenotype-specific risk such as *TP53* and atherosclerosis [19] and *TET2* and HF [13–15].

Knowledge gaps remain in our understanding of CHIP in patients with high cardiometabolic risk and existing CVD. To date there have been discrepant findings for CHIP and obesity traits, with initial studies showing an inverse association between CHIP and body-mass index (BMI) [3] while data from the UK Biobank showed a positive association between CHIP and waist-to-hip ratio (WHR) and *TET2* CHIP and BMI [20]. Although *DNMT3A* is the most commonly mutated CHIP gene, *DNMT3A* CHIP confers a lower risk of myeloid neoplasm than CHIP variants in other genes and the presence of a single *DNMT3A* variant is a favorable prognostic factor in the CH risk score (CHRS) [21]. Therefore, *DNMT3A* variants may not be the strongest drivers of cardiometabolic risk, particularly given that *DNMT3A* mutations were not associated with HF in a study of over 50,000 individuals [13] and specifically non-*DNMT3A* CHIP was associated acute kidney injury (AKI) and lack of recovery from AKI [22]. To date, the majority of literature on the cardiovascular association of CHIP has been derived from large epidemiologic cohorts, creating a knowledge gap for the impact of CHIP in high-risk complex patient populations with a high prevalence of CAD. Further, many studies have only treated CHIP as a binary variable without gene-specific analyses which may reveal distinct risk associations. Therefore, given the evolving CVD phenotypic associations, we sought to identify novel findings of CHIP, large CHIP and mutations in genes other than *DNMT3A* (non-*DNMT3A*) and cardiometabolic traits and to assess further validation for CVD events, HF and AF in a high-risk population. Here we leverage whole exome sequencing (WES) to explore the role of CHIP as an emerging biomarker for intermediate cardiometabolic risk factors and prevalent and incident CVD in a medically complex, but clinically relevant cohort of patients referred for cardiac catheterization.

## Methods

### Study population

The CATHGEN biorepository is comprised of 9334 individuals who underwent cardiac catheterization at Duke University Medical Center (Durham, NC) between January 1^st^, 2001 and December 31^st^, 2010 [23]. Femoral arterial blood samples were collected at the time of catheterization and whole blood was stored in ethylenediaminetetraacetic acid (EDTA) tubes. All study participants gave written informed consent for participation and use of their stored biospecimens for future use. The study was approved by the Duke University Institutional Review Board. For the present work, genetic sequencing data was generated in 2017 from samples collected at the time of enrollment in CATHGEN and accessed 27/08/2019. In the context of this ethical approval of retrospective work, the authors directly analyzing genetic and clinical data had access to information that could identify individual participants after data collection.

Demographics and comorbidities were collected through medical record review at study enrollment, and yearly follow-up was conducted for events and vital status through 2020. These data were supplemented with electronic health records, including International Classification of Diseases, Ninth Revision (ICD-9) and Tenth Revision (ICD-10) codes between January 1^st^, 2001 and August 31^st^, 2020 and accessed for research purposes 01/11/2020. Patients with a diagnosis of hematologic malignancy prior to or within 6 months of study enrollment were excluded for this study (n = 99, S1 Table in S2 File, “Prevalent Hematologic Malignancy” phenotype).

### Whole exome sequencing and somatic variant calling

WES was performed by Regeneron Genetics on DNA extracted from EDTA whole blood using the HiSeq 2500 platform (Illumina, San Diego, California). Following genomic sequence alignment and quality control, the resultant BAM files were used for somatic variant calling using the GATK [24] Mutect2 pipeline on the Terra platform [25,26]. A panel of normals (PON) created from 40 young, healthy participants, free from obstructive CAD, HF, hypertension or diabetes was used to eliminate sequencing artifacts. Functional annotation was performed for identified somatic variants and output into a variant call format (VCF) file. VCF files were parsed to regions of interest in 68 previously described CHIP genes and filtered to specific single-nucleotide variants (SNVs) and indels (S2 Table in S2 File).

Based on prior authoritative literature and consensus, CHIP was defined as presence of a variant at a VAF ≥ 2% and large CHIP clones at a VAF > 10% [27]. An experienced hematopathologist performed manual curation and review to refine the final variant call set. Additional details on sequencing and variant calling details can be found in the Supplemental Methods in S1 File. After WES quality control, 8469 participants had high quality somatic variant calls and were included for analyses. For participants with variants in multiple CHIP genes identified, analyses are categorized by the largest CHIP clone.

### Clinical data and outcomes

Baseline cardiometabolic comorbidities were assessed by the enrolling physician and medical record review. Obesity was defined as BMI ≥ 30 kg/m^2^. Prevalent CAD was defined as a prior history of MI, coronary artery bypass surgery, coronary percutaneous intervention, or cardiac catheterization with the presence of more than one major epicardial coronary vessel with 50−74% stenosis or one vessel with ≥75% stenosis. Prevalent CAD, HF and AF diagnoses were supplemented by ICD-9 and ICD-10 codes within six months or prior to index catheterization. All-cause mortality was determined using the Social Security Death Index (SSDI), National Death Index (NDI) and follow-up calls through the Duke Information System for Cardiovascular Care; cause of death was available on a subset of participants. ICD-9 and ICD-10 codes for emergency room visits or hospitalizations between 2001 and 2020 were used to define incident outcomes: MI and HF hospitalization (S1 Table in S2 File, “Myocardial Infarction” and “Heart Failure/ Cardiomyopathy” phenotype, respectively). Subjects with a HF outcome within 30 days of their enrollment catheterization were excluded to avoid new diagnoses of acute HF secondary to MI [28]. Incident AF was defined by ICD-9 or ICD-10 codes (S1 Table in S2 File, “Atrial Fibrillation” phenotype).

### GRACE score

Although not all CATHGEN participants presented with acute coronary syndrome, given the high prevalence of baseline CAD (64.6%) in a cardiac catheterization cohort we used the Global Registry of Acute Coronary Events (GRACE) score to determine the prognostic significance of CHIP for overall mortality prediction in addition to a validated clinical score [29] (Supplemental Methods in S1 File).

### Statistical analyses

A summary of the analytic approach is shown in S3 Table in S2 File. Associations of age with CHIP and clone size were tested using two sample t-test. Associations of CHIP, large CHIP and specific CHIP genes and obesity were tested using logistic regression in univariate and multivariate models. Across all multivariate analyses the basic covariates adjusted for were: age, genetic ancestry, sex, smoking. Sensitivity models for obesity included adjustment for the non-cardiac Charlson Comorbidity Index (CCI) as a marker of frailty (Supplemental Methods in S1 File).

The primary analyses were for the association of CHIP and obesity as a key cardiometabolic risk factor. Additional exploratory analyses for prevalent disease including CAD, HF and AF were further adjusted for diabetes, BMI, hypertension and hyperlipidemia. Sensitivity models for prevalent CAD included statin therapy (available in 61.2% of participants). HF and AF models were additionally adjusted for prevalent CAD and history of malignancy given the known associations of chemotherapy exposure with both CHIP and cardiomyopathy [28], and AF was also adjusted for prevalent HF. Stratified sensitivity analyses tested the association between HF phenotypes and CHIP: HFpEF (LVEF≥50%) and HF with mildly reduced or reduced ejection fraction (HFrEF, LVEF<50%) using ANOVA and chi-square tests to test CHIP proportions in these groups compared to participants without HF [30]. These HF phenotype sensitivity analyses excluded participants with a history of congenital heart disease, valvular disease, heart transplant or end-stage renal disease requiring dialysis.

Associations of CHIP and time-to-overall mortality were tested using Cox proportional hazard models and compared to the GRACE score. Fine-Gray competing risk regression models were used to test associations for incident outcomes with overall mortality as a competing risk: composite of MI or CV death, HF hospitalization and AF. Incident outcome analyses were adjusted for the same covariates as above including prevalent CAD, HF and AF. Given the long length of follow-up sensitivity analyses also truncated follow-up duration at ten years.

For each clinical outcome of interest, analyses were performed to test associations with the presence of any CHIP variant and large CHIP (VAF > 10%). To determine gene-specific risk, the most frequently mutated CHIP genes: *DNMT3A*, *TET2*, *ASXL1* and non-*DNMT3A* CHIP were also tested, requiring a minimum of ten cases or events for gene level analyses. Findings for overall CHIP with obesity and incident MI and CV death were considered the primary analysis and therefore significance was considered at p < 0.05 without adjustments for multiple comparisons and accordingly analyses for other outcomes and subgroups of gene types should be considered exploratory. Analyses were performed in R version 4.4.2.

## Results

### Baseline clinical characteristics and CHIP variants identified

Baseline participant characteristics are shown in Table 1. We identified an overall prevalence of CHIP of 5.0% with 463 CHIP variants in 427 unique participants, including 210 *DNMT3A*, 100 *TET2*, and 45 *ASXL1* variants (S1 Table in S2 File, S1a Fig). The median VAF across all CHIP variants was 13.2% (IQR 7.9–22.5%), including 289 large CHIP clones in 268 individuals (S1b Fig). As expected, participants with CHIP were older (mean 69.5 ± 10.3 years) than those without CHIP (60.8 ± 12.0 years, p < 2x10^-16^, S2 Fig). Individuals with large CHIP clones were on average 2.9 years older (mean 70.6 ± 9.7 years) than those with small CHIP clones (2% ≤ VAF ≤ 10%, 67.7 ± 11.0 years, p = 0.006).

**Table 1:** Baseline Participants Characteristics Stratified by CHIP Status.

	No CHIP	CHIP	p
n	8042	427	
Age (mean [SD], years)	60.8 (12.0)	69.5 (10.3)	<0.001
Sex, N female (%)	3030 (37.7)	189 (44.3)	0.007
Genetic Ancestry			0.045
European, N (%)	6095 (75.8)	346 (81.0)	
African, N (%)	1796 (22.3)	74 (17.3)	
Other, N (%)	151 (1.9)	7 (1.6)	
BMI (mean [SD], kg/m2)	30.1 (7.2)	28.90 (6.57)	0.001
Smoking History, N (%)	3832 (47.6)	197 (46.1)	0.6
Dyslipidemia, N (%)	4766 (59.3)	263 (61.6)	0.4
Diabetes, N (%)	2283 (28.4)	107 (25.1)	0.2
Hypertension, N (%)	5410 (67.3)	293 (68.6)	0.6

Characteristics of CATHGEN participants at time of enrollment are shown. Data presented as mean (standard deviation) or N (%). BMI, body-mass index (kg/m^2^).

Participants with CHIP were more often female (44.3%) than those without CHIP (37.7%, p = 0.007). *DNMT3A* CHIP carriers were mostly female (50.5%), whereas *TET2* and *ASXL1* CHIP carriers were predominantly male (55.8% and 81.3%, respectively). Participants with CHIP had a higher percentage of European ancestry (81.0%) than those without CHIP (75.8%, p = 0.045). Participants with CHIP also had a lower BMI (28.9 ± 6.6 kg/m^2^) than those without CHIP (30.1 ± 7.2 kg/m^2^ p = 0.001). There were no differences in history of smoking, dyslipidemia, diabetes or hypertension for overall CHIP, though 72.1% of *ASXL1* CHIP carriers had a history of smoking.

Thirty-two individuals had greater than one CHIP variant, five of which had two *TET2* variants, five had *TET2* and *DNMT3A* co-mutations and three had two *DNMT3A* variants (S3 Fig). Individuals with multiple CHIP variants were older (mean 72.5 ± 8.2 years, p = 0.045) and had greater history of MI (46.9% vs. 26.1%, p = 0.02) and coronary artery bypass surgery (46.9% vs. 20.5%, p = 0.001) than those with one CHIP variant.

### CHIP is associated with prevalent cardiometabolic disease

CHIP and large CHIP were inversely associated with obesity in univariate models and multivariate models (adjusted OR [aOR] CHIP 0.79 [95% CI 0.64–0.98], p = 0.03; aOR large CHIP 0.76 [95% CI 0.57–0.99], p = 0.04, S4 Fig, S5 Table in S2 File). Non-*DNMT3A*, *DNMT3A* and *ASXL1* CHIP were inversely associated with obesity in univariate models, but the relationship was attenuated in multivariate models. In sensitivity models adjusted for CCI the relationship between overall CHIP remained significant (aOR 0.80 [95% CI 0.65–0.99], p = 0.04, but the findings for large CHIP were attenuated (aOR 0.77 [95% CI 0.59–1.01], p = 0.07).

There were no significant associations with CHIP or large CHIP in univariate or multivariate analyses with prevalent CAD (S5 Table in S2 File). *ASXL1* CHIP was associated with CAD in univariate models (OR 5.36 [95% CI 2.16–17.89], p = 0.001), but was not significant in multivariate models (S5 Table in S2 File). There were no significant relationships in sensitivity models adjusting for baseline statin therapy.

**Fig 1:**
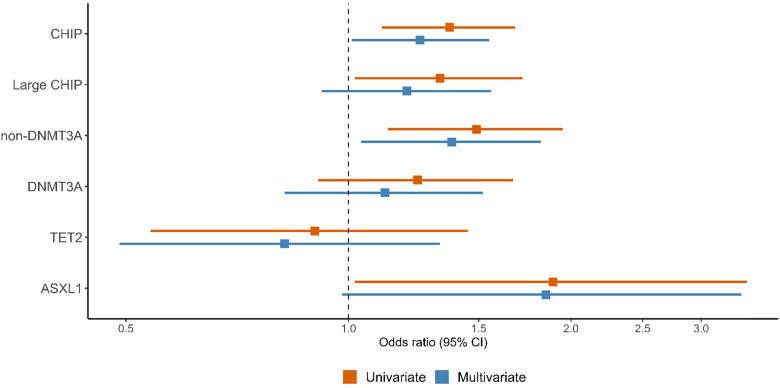
CHIP and non-*DNMT3A* CHIP Associate with Prevalent HF. The presence of any CHIP variant and non*-DNMT3A* was associated with higher odds of prevalent HF in CATHGEN. Multivariate models are adjusted for age, sex, genetic ancestry, smoking, diabetes, body-mass index, hypertension, hyperlipidemia, prevalent coronary artery disease and history of malignancy.

CHIP and large CHIP were associated with prevalent HF in univariate models, though only overall CHIP remained significant in multivariate analyses (aOR 1.25 [95% CI 1.01–1.55], p = 0.04, Fig 1, S5 Table in S2 File). Gene level analysis revealed that non-*DNMT3A,* and *ASXL1* CHIP carriers had a higher odds of prevalent HF in univariate analyses, only non-*DNMT3A* remained significant in multivariate analyses (aOR 1.38 [95% CI 1.04–1.82], p = 0.02). Sensitivity analyses of CHIP and HF phenotypes suggested the highest prevalence of CHIP in HFrEF (6.1%) vs. HFpEF (5.0%) vs. No HF (4.5%; ANOVA p = 0.04, chi-squared p = 0.04 for HFrEF vs. No HF, S5 Fig).

CHIP and large CHIP were associated with higher odds of prevalent AF in univariate models (OR CHIP 1.72 [95% CI 1.37–2.14], p = 1.8x10^-6^, OR large CHIP 1.75 [95% CI 1.32–2.30], 6.6x10^-5^), but the relationship was not significant in multivariate models (S6 Table in S2 File). Similarly, non-*DNMT3A*, *DNMT3A*, *TET2* and *ASXL1* CHIP were associated with higher odds of prevalent AF in univariate, but not multivariate models.

### CHIP associates with overall mortality in CATHGEN

The median follow-up time was 9.97 years (IQR 5.57–12.95 years) and there were 4197 total deaths. The presence of any CHIP variant and large CHIP were associated with time-to-death in univariate, but only large CHIP remained associated with time-to-death in multivariate models (aHR 1.17 [95% CI 1.01–1.36], p = 0.04, c-statistic = 0.675, S6 Table in S2 File, S6 Fig). The presence of CHIP in non-*DNMT3A*, *DNMT3A*, *TET2* and *ASXL1* were strongly associated with time-to-death in univariate models, only non-*DNMT3A* remained associated in multivariate models (aHR 1.31 [95% CI 1.12–1.54], p = 0.001, c-statistic = 0.676, S6 Table in S2 File, S6 Fig).

Given the high prevalence of baseline CAD in a cohort referred for catheterization we used the GRACE score to determine the prognostic value of CHIP in addition to a validated clinical score for predicting mortality. Mean GRACE score was higher in participants with CHIP, non-*DNTM3A* and large CHIP (92.0 ± 21.2, 93.1 ± 21.9 and 94.5 ± 20.7 points, respectively) versus those without CHIP (77.4 ± 22.0 points). The GRACE Score was strongly associated with mortality (aHR 1.02 [95% CI 1.01–1.02], p < 2.0x10^-16^, c-statistic = 0.682), where an increase in HR is associated with the risk associated with every additional GRACE score point. The addition of either large CHIP or non-*DNMT3A* CHIP to the GRACE score did not improve the c-statistic. In sensitivity models truncated at ten years, non-*DNMT3A* CHIP remained associated with time-to-death, but large CHIP was not. There were no significant differences in the predictive capability of the GRACE score or addition of non-*DNMT3A* CHIP at ten years.

### CHIP associates with incident cardiovascular disease

Over a median follow-up of 9.50 years (IQR 5.02–12.74 years), a total of 2286 participants suffered a composite outcome of time-to-first incident MI or CV death. While overall CHIP was not associated with the composite CV outcome, large CHIP clones were associated in univariate models (HR 1.37 [95% CI 1.11–1.68], p = 0.003) but this relationship was attenuated in multivariate models (aHR 1.21 [95% CI 1.01–1.44], p = 0.056, S7 Table in S2 File), suggesting the importance of clinical confounders, particularly age and cardiometabolic comorbidities. Similarly non-*DNMT3A* and *ASXL1* CHIP were associated with higher risk of the composite CV outcome in univariate models but were no longer significant in multivariate models (S7 Table in S2 File).

**Fig 2:**
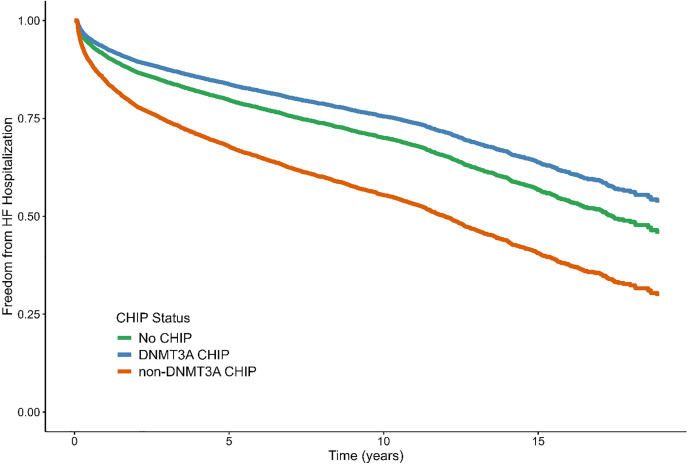
Non-*DNMT3A* and *DNMT3A* CHIP Associate with Incident HF Hospitalization. Adjust Kaplan-Meier curves for incident HF Hospitalization. Non-*DNMT3A* CHIP was associated with increased risk of incident HF hospitalization, whereas *DNMT3A* was associated with lower risk. Fine-Gray models are adjusted for age, sex, ancestry, smoking, diabetes, hypertension, hyperlipidemia, body-mass index, prevalent coronary artery disease, HF and atrial fibrillation with overall mortality as a competing risk. HF, heart failure.

Over a median follow-up of 8.08 years (IQR 3.21–12.00 years), there were 2541 participants with incident HF hospitalization. There were no significant associations for overall CHIP or large CHIP in univariate or multivariate models for incident HF hospitalization. Non-*DNMT3A* and *DNMT3A* CHIP were associated with incident HF hospitalization in both univariate and multivariate models, but with opposite direction of effect. Non-*DNMT3A* CHIP was associated with higher risk of HF hospitalization (aHR 1.29 [95% CI 1.02–1.63], p = 0.03), whereas the presence of *DNMT3A* CHIP was associated with lower risk of incident HF hospitalization (aHR 0.65 [95% CI 0.48–0.88], p = 0.005, S7 Table in S2 File, Fig 2). Non-*DNMT3A* and *DNMT3A* CHIP remained associated with time-to-HF hospitalization in sensitivity models truncated at ten years.

After restricting the population to participants without prevalent AF, there were 1398 subjects with incident AF over a median follow-up of 8.13 years (IQR 3.40–11.96 years). There were no significant associations with CHIP or large CHIP for time-to-incident AF diagnosis. Non-*DNMT3A* and *ASXL1* CHIP were associated with time-to-incident AF in univariate models, but only *ASXL1* CHIP remained associated in multivariate models (aHR 2.15 [95% CI 1.15–4.04], p = 0.02, S7 Table in S2 File, S7 Fig). *ASXL1* CHIP remained associated with time-to-AF diagnosis in sensitivity models truncated at ten years. Given the limited power in this sub-group analysis these findings should be considered exploratory and hypothesis-generating for future dedicated studies of CHIP and AF. Significant findings across analyses are shown in Table 2.

**Table 2:** Summary of Significant Associations of CHIP with Prevalent and Incident Cardiometabolic Disease in CATHGEN.

	Prevalent Outcomes				Incident Outcomes			
Gene	Obesity	CAD	HF	AF	Mortality	MI or CV death	HF Hospitalization	AF
CHIP	↓ / ↓	–	**↑** / **↑**	**↑** / -	**↑** / -	–	–	–
Large CHIP	↓ / ↓	–	**↑** / -	**↑** / -	**↑** / **↑**	**↑** / -	–	–
non-*DNMT3A*	↓ / -	–	**↑** / **↑**	**↑** / -	**↑** / **↑**	**↑** / -	**↑** / **↑**	**↑** / -
*DNMT3A*	↓ / -	–	–	**↑** / -	**↑** / -	–	↓ / ↓	–
*TET2*	–	–	–	**↑** / -	**↑** / -	–	–	–
*ASXL1*	↓ / -	**↑** / -	**↑** / -	**↑** / -	**↑** / -	**↑** / -	**↑** / -	**↑** / **↑**

Summary of significant associations for prevalent and incident outcomes analyzed. The first arrow indicates the direction of significant univariate associations, and the second arrow indicates significant multivariate associations; “-”, indicates no significant association. Downward pointing arrows with blue shading indicate an inverse association between CHIP and the outcome of interest, while upward pointing arrows with red shading indicate a positive association. CAD, coronary artery disease; CHIP, clonal hematopoiesis of indeterminate potential; HF, heart failure; AF, atrial fibrillation; MI, myocardial infarction; CV, cardiovascular.

## Discussion

**Fig 3:**
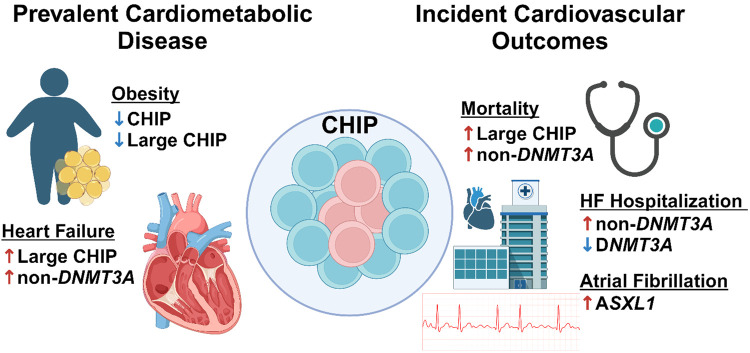
Central illustration. CHIP associates with prevalent and incident cardiometabolic disease in CATHGEN. The present work shows novel and confirmatory associations for CHIP including obesity, prevalent HF, mortality, HF hospitalization and atrial fibrillation in a cohort of patients referred for cardiac catheterization. CHIP, clonal hematopoiesis of indeterminate potential; HF, heart failure. Created in BioRender.

For the first time to date in a large, single-center study of 8469 high-risk participants referred for cardiac catheterization, we observed pleiotropic effects of large clones and non-*DNMT3A* CHIP on cardiometabolic traits and CV outcomes (Fig 3). We identified associations of CHIP with obesity, prevalent HF and incident HF hospitalization and AF. Additionally, we recapitulated the known age and mortality associations of CHIP. This study expands the existing CHIP literature on the effect of CHIP across cardiometabolic traits in a comorbid but clinically relevant cohort of high-risk participants referred for cardiac catheterization. Importantly, we show that though *DNMT3A* is the most frequently mutated CHIP gene, variants in non-*DNMT3A* genes drive cardiovascular risk associated with CHIP.

Although we expected to find a greater CHIP prevalence in this cohort enriched for CVD, we found a similar prevalence (5.0%) to previously published population-based CHIP cohorts [6,7,9,31], but lower than in a recent analysis of five TIMI trials (8.2%) with high baseline CVD [32]. CHIP prevalence in our study was lower than in recent work with a prevalence of 18.4% in 1142 individuals referred for cardiac catheterization at Vanderbilt University Medical Center, however Heimlich et al. used a targeted sequencing approach, with higher sensitivity than the WES used here which may miss the detection of small clones [33]. With respect to the association between CHIP and demographic characteristics, we found higher prevalence of CHIP in female participants in our cohort; this is discordant with the initial 2014 work by Jaiswal et al., where men had a higher odds of carrying a CHIP mutation [3]. However, our findings are similar to data from other cohorts showing that the effect on sex depends on the driver gene, where there is an increased prevalence of *DNMT3A* mutations in females, but other CHIP driver genes including *TET2* and *ASXL1* are more prevalent in males. We replicated similar findings as have been published between smoking history and *ASXL1* CHIP, but there was no difference for smoking history and overall CHIP [6,7,34,35].

Interestingly, with respect to cardiometabolic risk factors, we observed an inverse association between CHIP, large CHIP and obesity. To date, there have been conflicting findings between CHIP and obesity. For example, Jaiswal et al. found an inverse relationship between CHIP and BMI [3], however in 8709 postmenopausal women from the Women’s Health Initiative (WHI), normal and overweight BMI participants had a lower odds of *DNMT3A* and *TET2* CHIP than obese participants [36]. In 47,466 UK Biobank participants, CHIP was associated with higher waist-to-hip-ratio, though only *TET2* CHIP was associated with high BMI [20]. Though we have described for the first time the association of large CHIP clones and obesity, we observed no significant associations for gene-specific analyses and obesity. Conversely, obesity may exacerbate CHIP. Mice harboring *TET2* and *DNMT3A* CH crossed with genetically obese mice have shown expansion of CHIP clones and heightened levels of pro-inflammatory cytokines [20]. In our cohort there was a strong inverse relationship between age and BMI; this may reflect sarcopenia and frailty, as findings for overall CHIP remained significant even after adjusted for the CCI. There may be a complex and potentially parabolic relationship between CHIP and BMI, with higher prevalence of CHIP at both extremes of BMI. One limitation to this and many historic studies is the lack of availability of body composition measurements as more granular markers of obesity, muscle mass and sarcopenia. Further, survival bias may confound these results, whereby patients with CHIP and higher BMI may not have survived long enough to be included in the study. Future studies should further explore gene-specific metabolic underpinnings of the obesity paradox seen with CHIP in our present work and consider the role of CHIP in body composition, regional and organ-specific adiposity.

Interestingly, we did not identify significant associations for CHIP or large CHIP with prevalent CAD. The lack of association may be related to survival bias, the high prevalence of comorbidities and CAD in the CATHGEN cohort, including with the highly sensitive detection of CAD with invasive assessment of coronary lesions in the majority of patients studied. In a recent analysis of five TIMI trials, there was similar baseline prevalence of atherosclerotic CVD between those with and without CHIP and, similar to our observation here, a lower percentage with prior MI in those with CHIP [32]. Taken together, these findings support CHIP as a stronger risk marker for incident disease than prevalent cardiometabolic traits.

To the first time to our knowledge, we identified higher odds of prevalent HF in CHIP carriers in the CATHGEN cohort, with strong associations for non-*DNMT3A* variants. CHIP prevalence was greatest in those with HFrEF, while HFpEF prevalence was intermediate to those without HF. Here we provide key associations for CHIP and prevalent HF, even after adjustment for CAD, adding further support to the growing literature of pathophysiologic mechanisms linking CHIP and HF, which are at least partly independent of atherosclerosis. In chronic HF, neurohormonal activation triggers immune systemic activation, pathological tissue remodeling with detection of heightened levels of high-sensitivity C-reactive protein (hsCRP) and IL-6 [37]. Therefore, potential mechanisms of the findings observed here include heightened levels of systemic inflammation, which have been more clearly observed in non-*DNMT3A* CHIP, particularly *TET2* [10]. In recent work using ultradeep error-corrected sequencing, prevalence of *TET2* CH (VAF > 0.5%) was enriched in HFpEF cases compared to controls [15]. Concordant with prior studies, we also identified greater risk for incident HF hospitalization. Although not significant for overall CHIP, we found associations for non-*DNMT3A* CHIP and incident HF hospitalization.

Surprisingly, *DNMT3A* CHIP was associated with lower risk of incident HF hospitalization. This finding may reflect unmeasured protective confounders in participants with *DNMT3A* CHIP or may reflect distinct molecular mechanisms invoked by *DNMT3A* variants in contrast to other CHIP genes. Specifically, loss-of-function mutations in *DNMT3A* reduce global methylation and epigenetically reprogram HSC self-renewal, impairing cell differentiation [38]. The clonal dynamics of *DNMT3A* are observed to have more steady state growth with less inflammatory influence in contrast to other CHIP genes (e.g., *TET2*, *ASXL1*, *JAK2*) [10,39,40]. Although these pathobiological mechanisms may explain why *DNMT3A* variants have a more neutral impact on CVD, protective mechanisms that would explain this inverse relationship warrant further study, including in future prospective work. In a meta-analysis of over 56,000 participants, CHIP was associated with incident HF, specifically *ASXL1*, *TET2* and *JAK2* variants, but the authors did not find significant associations with *DNMT3A* CHIP [13]. Along with the results provided here this adds support that *DNMT3A* CHIP may not be a clear driver of HF risk. More recently, *TET2* CHIP, but not overall CHIP was associated with incident HFpEF-specific hospitalization in 8090 participants from the Jackson Health Study and WHI [14]. In murine models, *TET2* CH exacerbated features of HFpEF compared to wild-type bone marrow. Taken together, these findings suggest that variants in CHIP genes other than *DNMT3A* drive both prevalent and incident HF risk and ongoing work is needed to decipher gene and variant specific risk in clinical and pre-clinical research. Given the complex systemic and metabolic changes in HFpEF, the role of CHIP and its associated inflammatory should be assessed more deeply in future work.

Only large and non-*DNMT3A* CHIP were associated with overall mortality in CATHGEN after adjustment for relevant clinical covariates, however there was no improvement in risk prediction when added to the GRACE score [29]. The lack of association for overall CHIP with mortality suggests age and other clinical variables remain strong drivers of mortality in the broad, heterogenous definition of CHIP. Further, *DNMT3A* makes up the largest proportion (49.2%) of overall CHIP in our study yet is proving to not be a driver of inflammation and adverse effects. Therefore, *DNMT3A* may confound any signal between overall CHIP and mortality, particularly given the inverse associations described above for *DNMT3A* CHIP with HF hospitalization, a strong prognostic event. This work adds to the growing body of literature that *DNMT3A* variants are not strong drivers of cardiac or malignant disease, including a recent study of 194 patients with aortic stenosis where non-*DNMT3A* CHIP, but not overall CHIP was associated with mortality [41]. Unlike prior work, CHIP was not associated with time-to-incident MI or CV death in competing risk regression models in CATHGEN. Large CHIP and non-*DNMT3A*, including *ASXL1* were significant in univariate models though the relationship was attenuated in multivariate models. Interestingly, large CHIP was not associated with HF hospitalization even in univariate models, suggesting risk associated with clone size may be more specific for atherosclerotic phenotypes. These findings are in contrast to data from 424,651 UK Biobank participants, in whom CHIP was associated with incident CVD events (composite MI, CAD or revascularization, stroke or death) with strong associations for large CHIP variants in each gene assessed, including *ASXL1* [42]. In 13,129 individuals in the UK Biobank with established CVD, CHIP and large CHIP were associated with a primary outcome of CVD events, with *TET2* and spliceosome CHIP (*SF3B1*, *SRSF2*, *U2AF1*) having the strongest association with adverse outcomes [43]. In CATHGEN, *ASXL1* CHIP was associated with incident MI or CV death, as well as HF hospitalization in univariate, but not multivariate models. In 235 participants with established MDS, *ASXL1* variants were highly prevalent (40%) and associated with risk of vascular events [44]. Recently, the CHRS, a score developed and validated for risk of myeloid neoplasm where non-*DNMT3A* CHIP confers greater risk, has also been shown to be associated with overall and cardiovascular mortality [45]. The work presented here supports the need for ongoing mutation-specific analyses of CHIP-risk phenotypes and underlying mechanisms, particularly for non-*DNTM3A* CHIP.

We did not identify any significant findings between CHIP and prevalent AF risk, including when looking at large CHIP clones. Concordant with prior work, *ASXL1* CHIP was associated with risk of incident AF. A phenome-wide association study (PheWAS) found baseline CHIP to be associated with incident AF in the UK Biobank [8]. More recently, large *TET2* and *ASXL1* CHIP clones, but not any CHIP or *DNMT3A* CHIP were associated with incident AF in nearly 200,000 participants from the UK Biobank and Atherosclerosis Risk in Communities (ARIC) studies [17]. Further murine models of AF and *TET2* CHIP suggest the therapeutic potential of NLPR3 inflammasome blockade in arrhythmia [18]. Future studies should continue to explore the connection between arrhythmias, CHIP and dysregulated inflammation.

Despite multiple strengths, including long duration of follow-up in a comorbid cohort of diverse participants with ability for detailed EHR review at the individual-participant level, this work has limitations. First, as all participants had at least concern for baseline CVD prompting catheterization referral, the study sample from a cardiac catheterization biorepository limits the generalizability to a broader patient population. Despite this limitation, the heterogeneity in the cohort studied here may be more directly analogous to observations expected in real-world clinical practice for patients with prevalent CVD. Second, as a tertiary referral center, not all participants were followed in Duke University Health System long term. This limited incident data from ICD codes which are linked to inpatient and emergency room encounters at Duke. We recognize the study may suffer from survival bias for those individuals who may have had CHIP and survived to get catheterization. Third, WES is not the most sensitive method to detect small somatic clones which may not have been detected in this dataset and therefore contributes to an overall lower prevalence of CHIP in this compared to others using targeted sequencing methods. This could influence the rate of false negatives, impacting downstream analyses, however it should be noted that the majority of large CHIP studies to date have relied on sequencing technologies of similar sensitivity (WES or whole-genome sequencing) [6]. Further, our ability to analyze *JAK2*-specific associations in CATHGEN was limited due to the lower than expected number of *JAK2* V617F variants likely secondary to limited coverage of the exons of interest in this region. Finally, subgroup analyses for gene types are limited in power with decreasing prevalence and these and non-primary outcomes measures were not adjusted for multiple comparisons.

In summary, in a cohort of participants referred for cardiac catheterization, CHIP and large CHIP were inversely associated with obesity. CHIP and non-*DNMT3A* CHIP were associated with prevalent HF, even in patients with a history of malignancy or chemotherapy exposure. We also identified risk of mortality and incident outcomes for large, non-*DNMT3A* and *ASXL1* CHIP. These findings support concrete clinical implications for the cardiovascular care and risk stratification of patients with CHIP which requires a gene- and clone-size specific risk assessment. As carriers of large and non-*DNMT3A* appear to be at greatest risk for HF and adverse outcomes, aggressive preventive strategies, focused on cardiometabolic risk factors including antihypertensive and lipid lowering therapies, should be considered in these patients. Although, further work is warranted to understand the mechanistic underpinnings of the relationships of specific CHIP mutations, inflammation and cardiometabolic disease, utilization and refinement of CHIP as a CVD biomarker may allow for improved precision medicine approaches to patient care.

## Supporting information

S1 Fig Gene Breakdown and Violin Plot of Variant Allele Fraction for Top 8 CHIP Gene. A. Tree plot of top 8 most frequently mutated genes out of 463 total CHIP mutations (*DNMT3A* n = 210, *TET2* n = 100, *ASXL1* n = 45, *PPM1D* n = 20, *SF3B1* n = 17, *TP53* n = 16, *GNB1* n = 7, *JAK2* n = 6). B. Violin plot by Variant Allele Fraction (VAF) of top eight most frequently mutated genes in CATHGEN.
(TIF)

S2 Fig Age Associated with CHIP in CATHGEN. Participants with CHIP were older on average 8.7 years older than those without CHIP (mean 69.5 ± 10.3 years vs 60.8 ± 12.0 years, p < 2x10^-16^). There was a higher percentage of CHIP carriers by decade. The overall prevalence of CHIP in the cohort was 5.0%.
(TIF)

S3 Fig Co-mutation plot for CATHGEN participants with more than one CHIP variant. Waterfall plot of CATHGEN participants with more than one CHIP variant identified. *TET2* variants were most frequently identified in participants with more than one CHIP variant. Clones in individuals with more than one CHIP variant were larger (VAF 20.4% ± 13.3%) than clones in individuals with one CHIP variant (VAF 15.8% ± 11.5%).
(TIF)

S4 Fig Forest Plot for Association of CHIP and Obesity. CHIP and large CHIP were inversely associated with obesity in both univariate and multivariate models adjusted for age, sex, ancestry and history of smoking. Non-*DNMT3A*, *DNMT3A* and *ASXL1* CHIP were associated with lower odds of obesity in univariate but not multivariate models.
(TIF)

S5 Fig Prevalence of CHIP by HF Phenotype. Sensitivity analyses tested the association between heart failure (HF) phenotypes and CHIP. No HF (N = 4531, 4.5% CHIP); heart failure with preserved ejection fraction, HFpEF (EF ≥ 50%, N = 726, 5.0% CHIP); heart failure with reduced or mildly reduced ejection fraction, HFrEF (EF < 50%, N = 928, 6.1% CHIP). ANOVA p = 0.04, chi-squared p = 0.04 for HFrEF vs. No HF.
(TIF)

S6 Fig Large CHIP and non-*DNMT3A* CHIP associate with time-to-overall mortality in CATHGEN. Adjusted Kaplan-Meier curves for the association of large CHIP clones (VAF > 10%, Panel A). and non-*DNMT3A* CHIP variants (Panel B). Multivariate cox models are adjusted for age, sex, ancestry, smoking, diabetes, hypertension, hyperlipidemia, body-mass index, prevalent coronary artery disease, heart failure and atrial fibrillation.
(TIF)

S7 Fig *ASXL* CHIP Associates with Risk of Incident Atrial Fibrillation in CATHGEN. Adjusted Kaplan-Meier curve for *ASXL1* CHIP and incident atrial fibrillation. Models are adjusted for age, sex, ancestry, smoking, diabetes, hypertension, hyperlipidemia, body-mass index, prevalent coronary disease and heart failure.
(TIF)

S1 File Supplemental Material. This file includes Supplemental Methods.
(DOCX)

S2 File **S1–S7 Supplemental Tables.** This file includes Supplemental Tables. **S1 Table.** Clinical ICD Codes Used to Define Prevalent and Incident Outcomes. Table of all ICD 9 and 10 codes used to define prevalent and incident outcomes from electronic health record data in CATHGEN. ICD, International Classification of Diseases. **S2 Table.** Transcripts, genes and variants used for CHIP calling. Table of 68 genes, relevant variants and transcripts used for variant calling. **S3 Table.** Statistical Analysis Approach. Summary of the statistical analysis approach for prevalent and incident outcomes. Basic covariates = Age, Sex, Ancestry, Smoking; *included in sensitivity model for CAD; HF phenotypes include HF with reduced ejection fraction (HFrEF, EF < 50%) and HF with preserved ejection fraction (HFpEF, EF ≥ 50%); BMI, body mass index; CAD, coronary artery disease; HF, heart failure; MI, myocardial infarction; CV, cardiovascular; ANOVA, analysis of variance; PH, proportional hazards; CRR, competing risk reduction. **S4 Table.** 463 CHIP variants identified in CATHGEN. Variant level details for the 463 CHIP variants identified in 427 CATHGEN participants. **S5 Table.** Associations of CHIP with prevalent cardiometabolic disease. Overall, large CHIP and gene-specific analysis for associations of CHIP with prevalent cardiometabolic disease. *Associations for obese vs. non-obese participants defined as body-mass index (BMI) ≥30 mg/kg2. Multivariate analyses are adjsuted for age, sex, ancestry and smoking. ^Multivariate analyses for coronary artery disease (CAD) are adjusted for age, sex, ancestry, smoking, diabetes, BMI, hypertension and hyperlipidemia. CAD sensitivity model included statin use for participants with medication data available. #Multivariate analyses for heart failure (HF) and atrial fibrillation (AF) are adjusted for age, sex, ancestry, smoking, diabetes, BMI, hypertension, hyperlipidemia, prevalent CAD and history of malignancy. AF models are additionally adjusted for prevalent HF. **S6 Table.** Associations of CHIP and time-to-overall mortality. Overall, large CHIP and gene-specific analysis for associations of CHIP and time-to-overall mortality in CATHGEN. *Cox proportional hazard (PH) models for time-to-overall mortality. Multivariate model includes age, sex, ancestry, smoking, diabetes, hyperlipidemia, hypertension, obesity, prevalent coronary artery disease, heart failure and atrial fibrillation. **S7 Table.** Fine-Gray associations of CHIP and incident clinical outcomes. Competing risk regression models for incident clinical outcomes with overall mortality as a competing risk. *Multivariate models for incident cardiovascular (CV) outcomes are adjusted for age, sex, ancestry, smoking, diabetes, hypertension, hyperlipidemia, body-mass index, prevalent coronary disease (CAD) and heart failure (HF). Models for MI or CV death and HF hospitalization are also adjusted for prevalent atrial fibrillation.
(XLSX)

## Abbreviations

CHIP: clonal hematopoiesis of indeterminate potential
VAF: variant allele fraction
CATHGEN: Catheterization Genetics
CAD: coronary artery disease
MI: myocardial infarction
CV: cardiovascular
CVD: cardiovascular disease
HF: heart failure
AF: atrial fibrillation.
