# Supplementary figures and images for: Clonal hematopoiesis associates with prevalent and incident cardiometabolic disease in a cardiac catheterization cohort
Source: PLoS One. 2026 Feb 10;21(2):e0339491. doi: 10.1371/journal.pone.0339491 (PMC12890114; doi:10.1371/journal.pone.0339491)

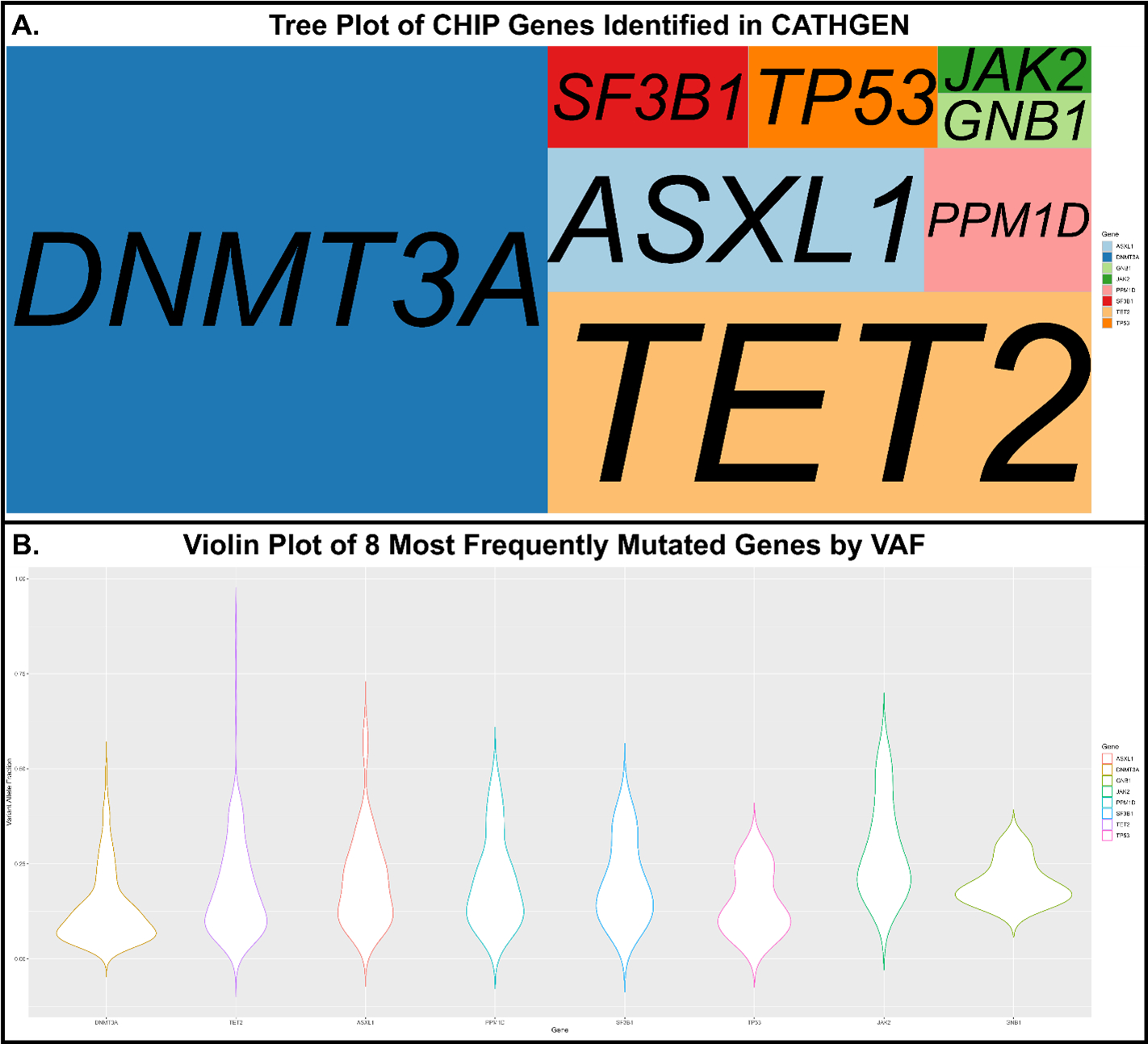

Supplement: S1 Fig — A. Tree plot of top 8 most frequently mutated genes out of 463 total CHIP mutations (DNMT3A n = 210, TET2 n = 100, ASXL1 n = 45, PPM1D n = 20, SF3B1 n = 17, TP53 n = 16, GNB1 n = 7, JAK2 n = 6). B. Violin plot by Variant Allele Fraction (VAF) of top eight most frequently mutated genes in CATHGEN. (TIF) [file pone.0339491.s001.tif]

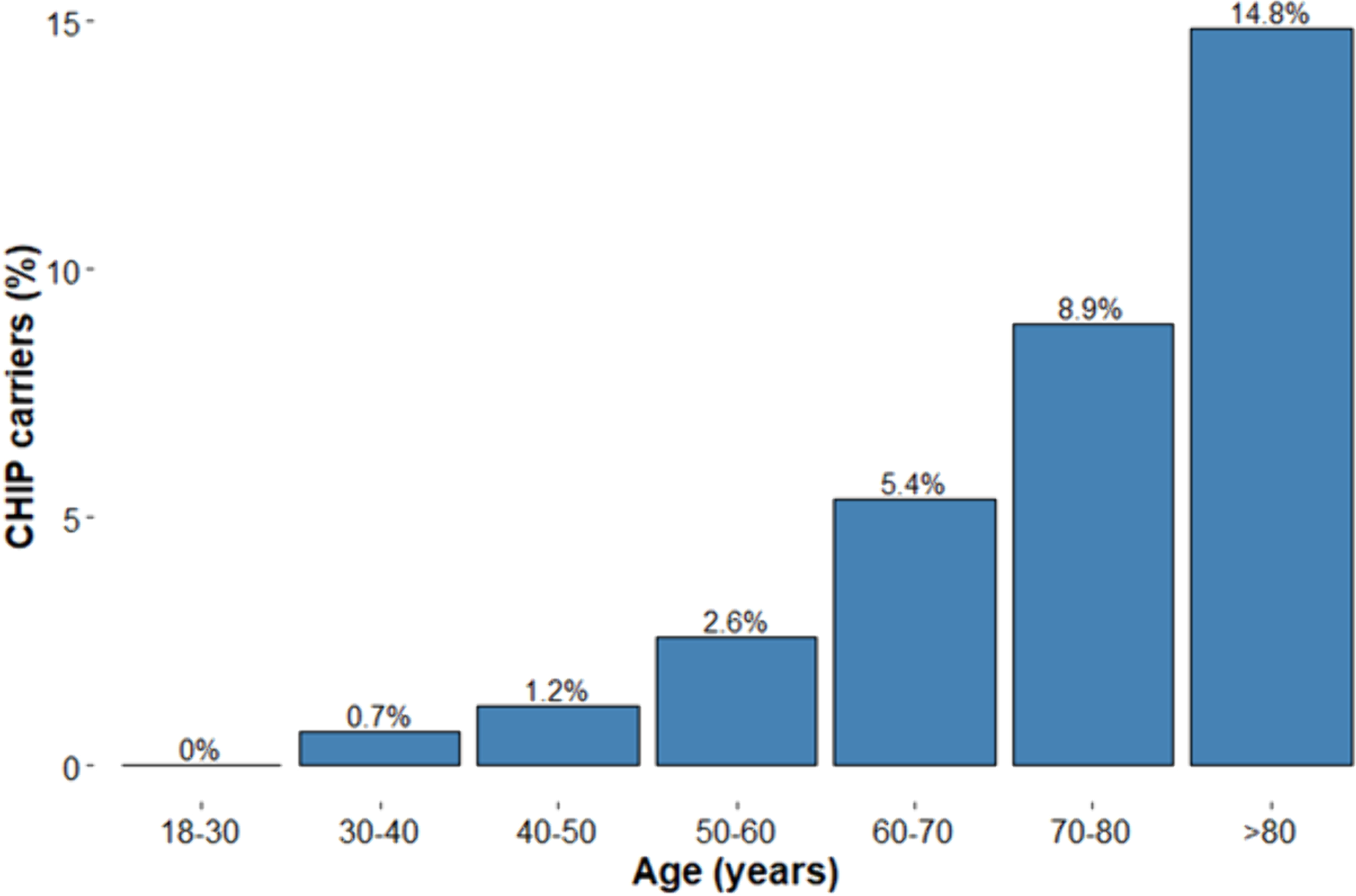

Supplement: S2 Fig — Participants with CHIP were older on average 8.7 years older than those without CHIP (mean 69.5 ± 10.3 years vs 60.8 ± 12.0 years, p < 2x10^-16^). There was a higher percentage of CHIP carriers by decade. The overall prevalence of CHIP in the cohort was 5.0%. (TIF) [file pone.0339491.s002.tif]

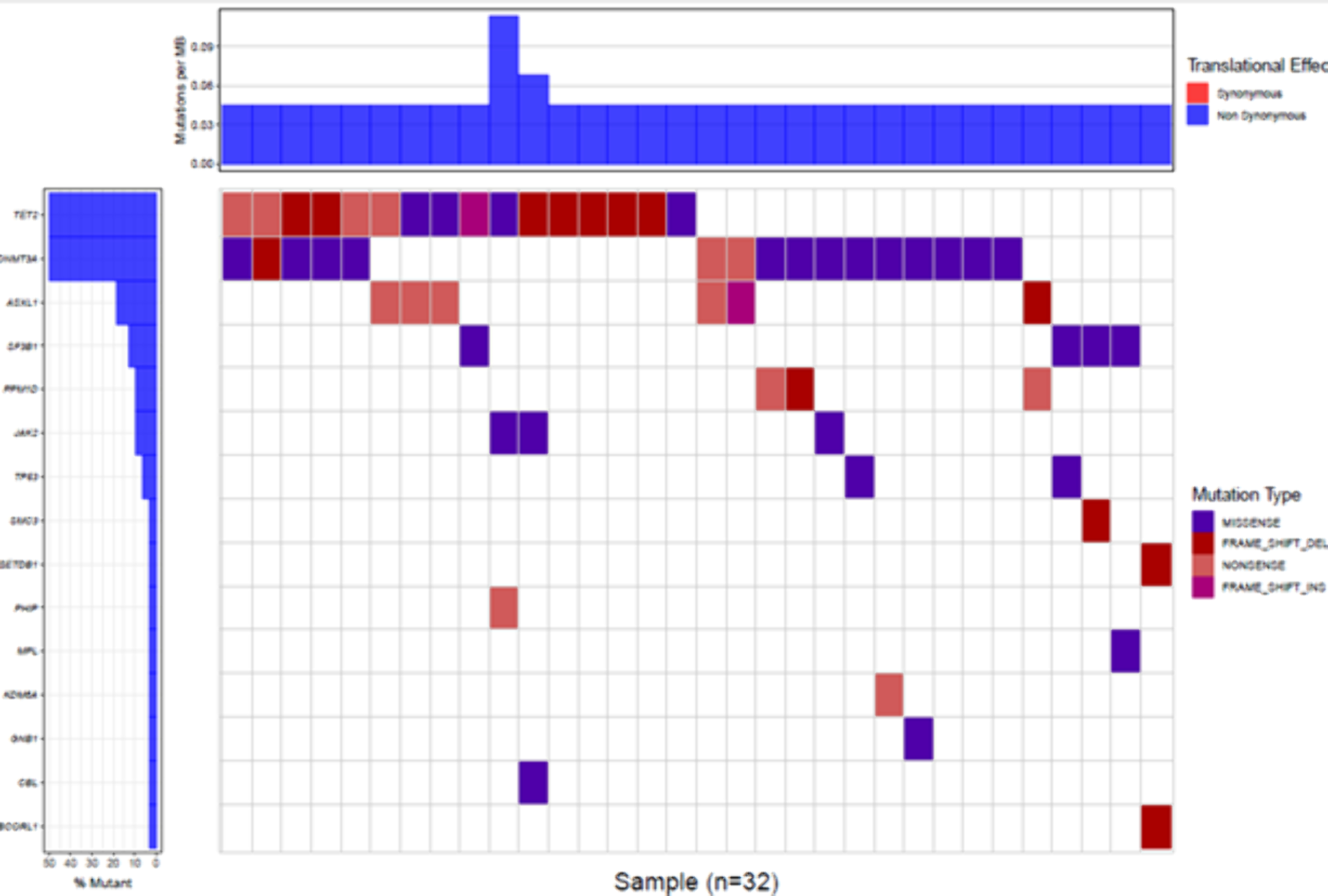

Supplement: S3 Fig — Waterfall plot of CATHGEN participants with more than one CHIP variant identified. TET2 variants were most frequently identified in participants with more than one CHIP variant. Clones in individuals with more than one CHIP variant were larger (VAF 20.4% ± 13.3%) than clones in individuals with one CHIP variant (VAF 15.8% ± 11.5%). (TIF) [file pone.0339491.s003.tif]

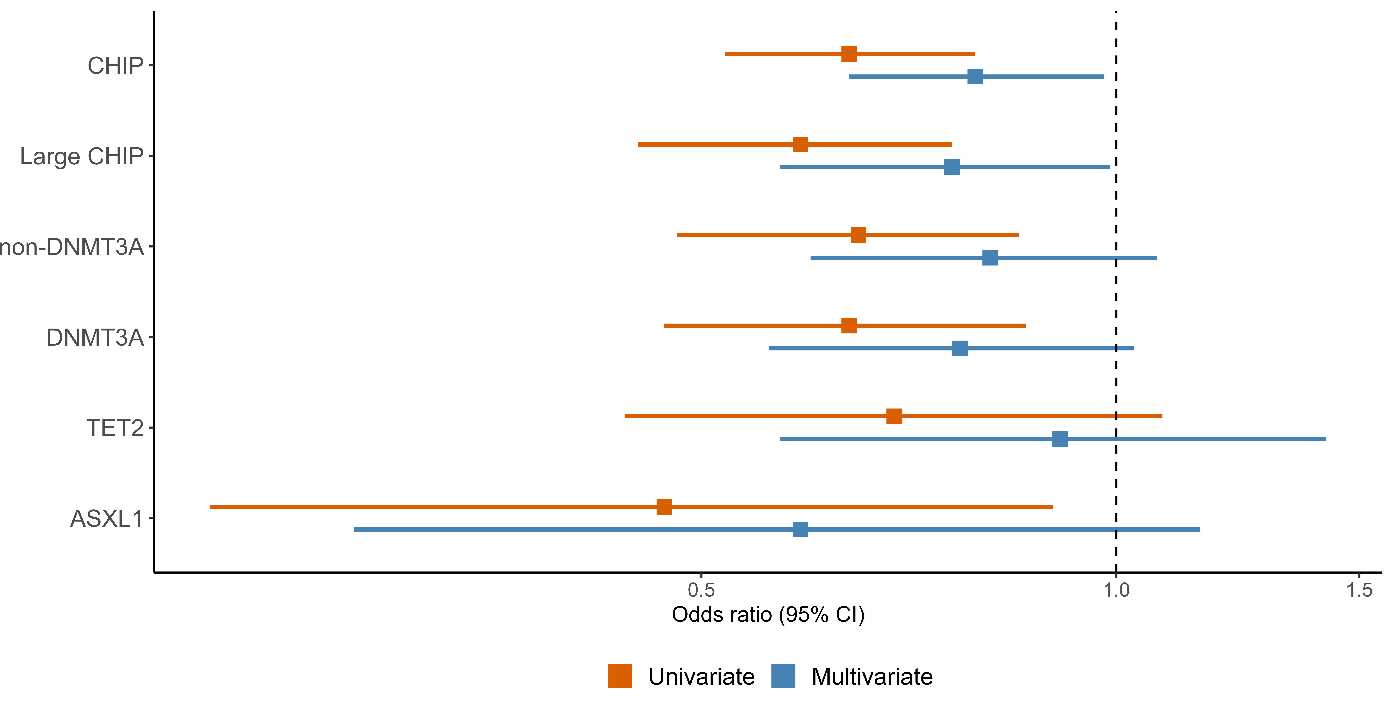

Supplement: S4 Fig — CHIP and large CHIP were inversely associated with obesity in both univariate and multivariate models adjusted for age, sex, ancestry and history of smoking. Non-DNMT3A, DNMT3A and ASXL1 CHIP were associated with lower odds of obesity in univariate but not multivariate models. (TIF) [file pone.0339491.s004.tif]

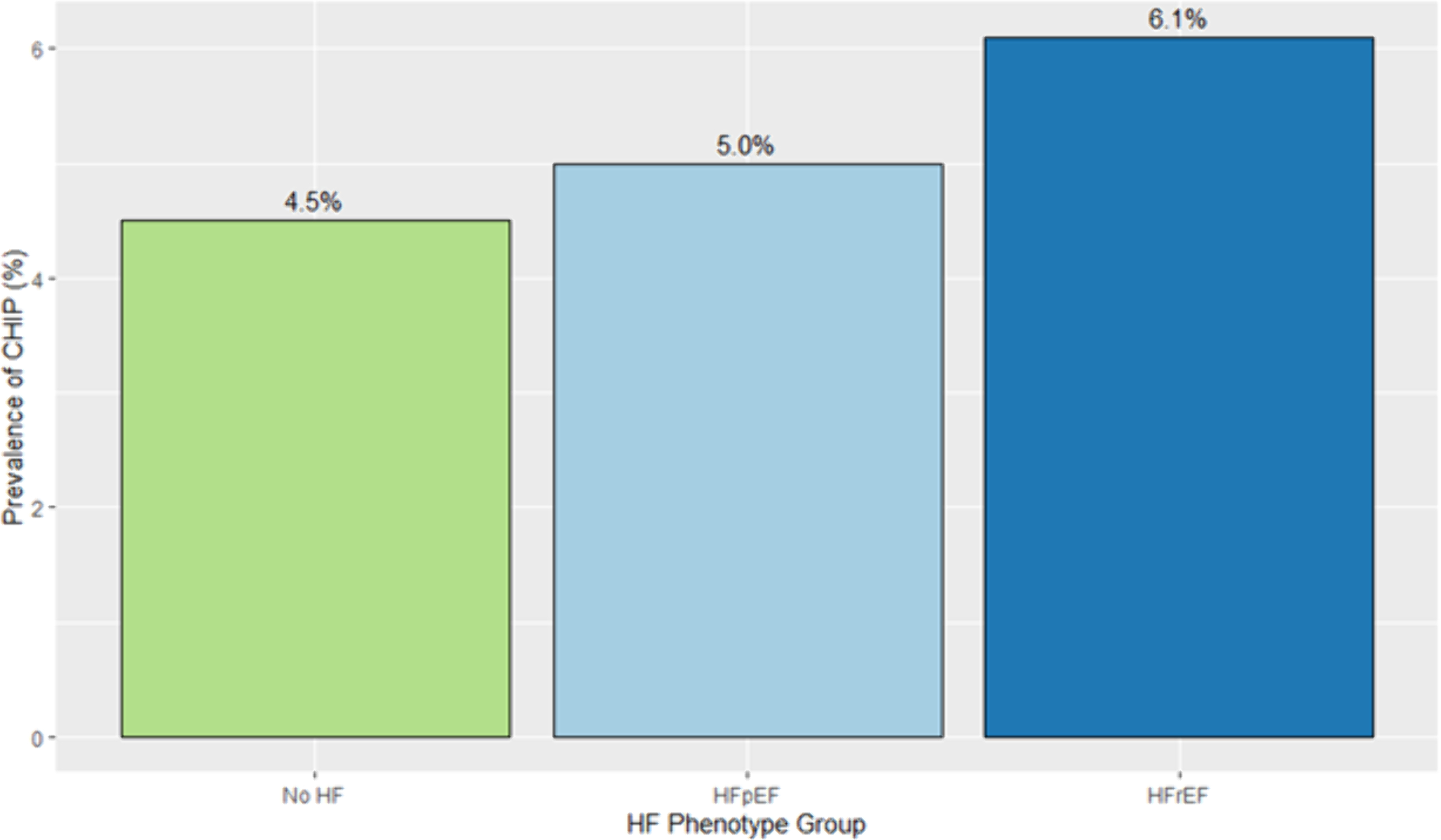

Supplement: S5 Fig — Sensitivity analyses tested the association between heart failure (HF) phenotypes and CHIP. No HF (N = 4531, 4.5% CHIP); heart failure with preserved ejection fraction, HFpEF (EF ≥ 50%, N = 726, 5.0% CHIP); heart failure with reduced or mildly reduced ejection fraction, HFrEF (EF < 50%, N = 928, 6.1% CHIP). ANOVA p = 0.04, chi-squared p = 0.04 for HFrEF vs. No HF. (TIF) [file pone.0339491.s005.tif]

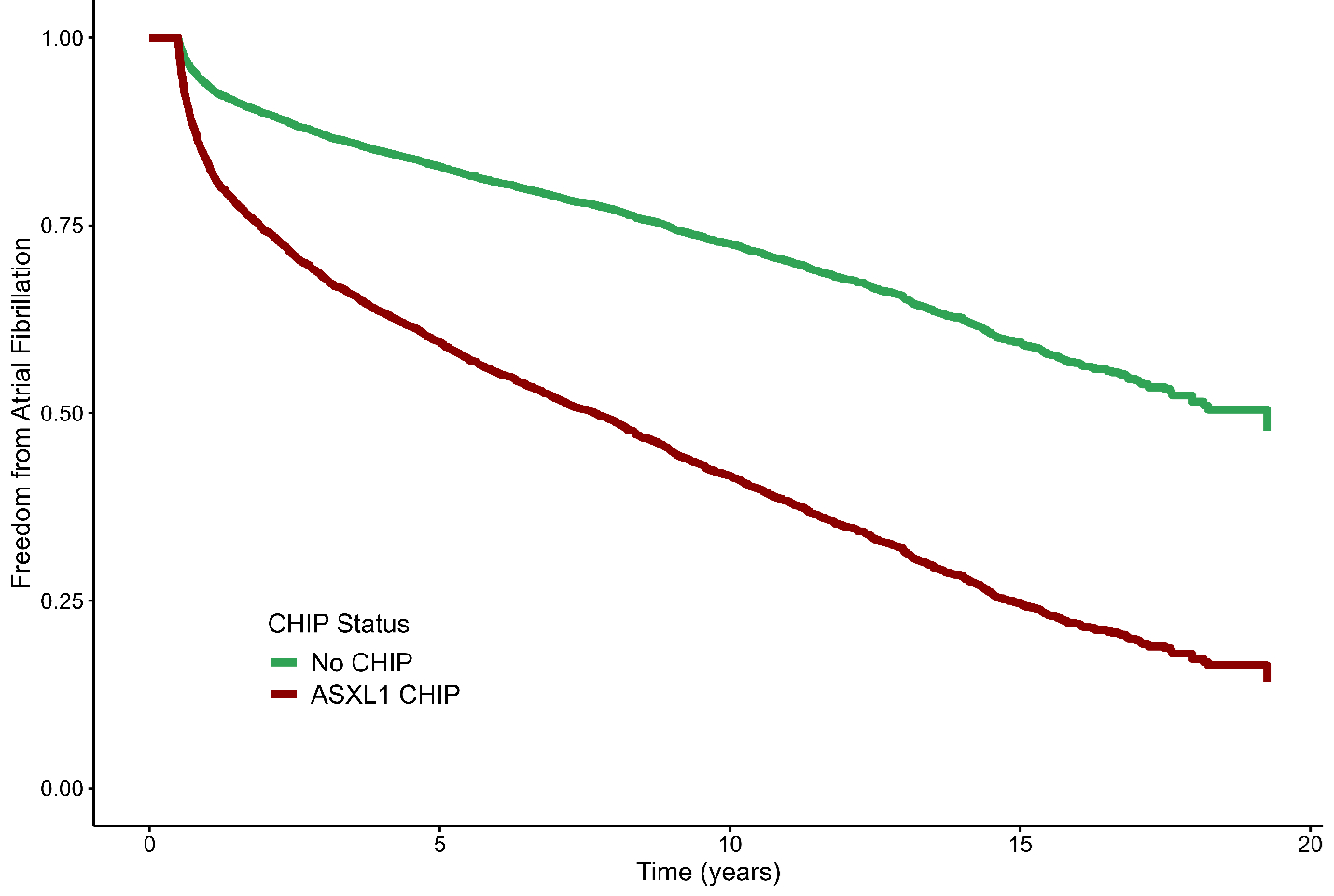

Supplement: S7 Fig — Adjusted Kaplan-Meier curve for ASXL1 CHIP and incident atrial fibrillation. Models are adjusted for age, sex, ancestry, smoking, diabetes, hypertension, hyperlipidemia, body-mass index, prevalent coronary disease and heart failure. (TIF) [file pone.0339491.s007.tif]
